# Diagnosis and detection of bone fracture in radiographic images using deep learning approaches

**DOI:** 10.3389/fmed.2024.1506686

**Published:** 2025-01-24

**Authors:** Theyazn Aldhyani, Zeyad A. T. Ahmed, Bayan M. Alsharbi, Sultan Ahmad, Mosleh Hmoud Al-Adhaileh, Ahmed Hassan Kamal, Mohammed Almaiah, Jabeen Nazeer

**Affiliations:** ^1^Applied College, King Faisal University, Al-Ahsa, Saudi Arabia; ^2^Department of Computer Science, Dr. Babasaheb Ambedkar Marathwada University, Aurangabad, India; ^3^Department of Information Technology, College of Computers and Information Technology, Taif University, Taif, Saudi Arabia; ^4^Department of Computer Science, College of Computer Engineering and Sciences, Prince Sattam Bin Abdulaziz University, Alkharj, Saudi Arabia; ^5^School of Computer Science and Engineering, Lovely Professional University, Phagwara, Punjab, India; ^6^Deanship of E-Learning and Distance Education and Information Technology, King Faisal University, Al-Ahsa, Saudi Arabia; ^7^Department of Orthopedic and Trauma, College of Medicine, King Faisal University, Al-Ahsa, Saudi Arabia; ^8^King Abdullah the II IT School, The University of Jordan, Amman, Jordan

**Keywords:** deep learning, artificial intelligence, radiographic images, bone fractures, diagnosis

## Abstract

**Introduction:**

Bones are a fundamental component of human anatomy, enabling movement and support. Bone fractures are prevalent in the human body, and their accurate diagnosis is crucial in medical practice. In response to this challenge, researchers have turned to deep-learning (DL) algorithms. Recent advancements in sophisticated DL methodologies have helped overcome existing issues in fracture detection.

**Methods:**

Nevertheless, it is essential to develop an automated approach for identifying fractures using the multi-region X-ray dataset from Kaggle, which contains a comprehensive collection of 10,580 radiographic images. This study advocates for the use of DL techniques, including VGG16, ResNet152V2, and DenseNet201, for the detection and diagnosis of bone fractures.

**Results:**

The experimental findings demonstrate that the proposed approach accurately identifies and classifies various types of fractures. Our system, incorporating DenseNet201 and VGG16, achieved an accuracy rate of 97% during the validation phase. By addressing these challenges, we can further improve DL models for fracture detection. This article tackles the limitations of existing methods for fracture detection and diagnosis and proposes a system that improves accuracy.

**Conclusion:**

The findings lay the foundation for future improvements to radiographic systems used in bone fracture diagnosis.

## 1 Introduction

Bones are a vital component of human anatomy, enabling movement and providing structural support. Bone fractures, classified as either partial or complete, are disruptions in bone continuity. Tibial fractures are the most common type, particularly affecting children, athletes, and the elderly, and present significant diagnostic challenges. Consequently, a rapid and accurate diagnosis is critical for enhancing the efficiency of the healing process (1).

X-ray, computed tomography (CT), and magnetic resonance imaging (MRI) are the predominant imaging modalities used for various conditions, particularly in fracture diagnosis. Among these, X-ray is the most widely used and accessible diagnostic tool, in which the targeted body region is exposed to X-ray radiation. Despite limitations in image quality, X-rays are sufficient for detecting fractures (2, 3). Bone fractures can result from accidents or other factors and require prompt care. Orthopedic surgeons typically analyze X-rays to identify fractures.

**Figure 1:**
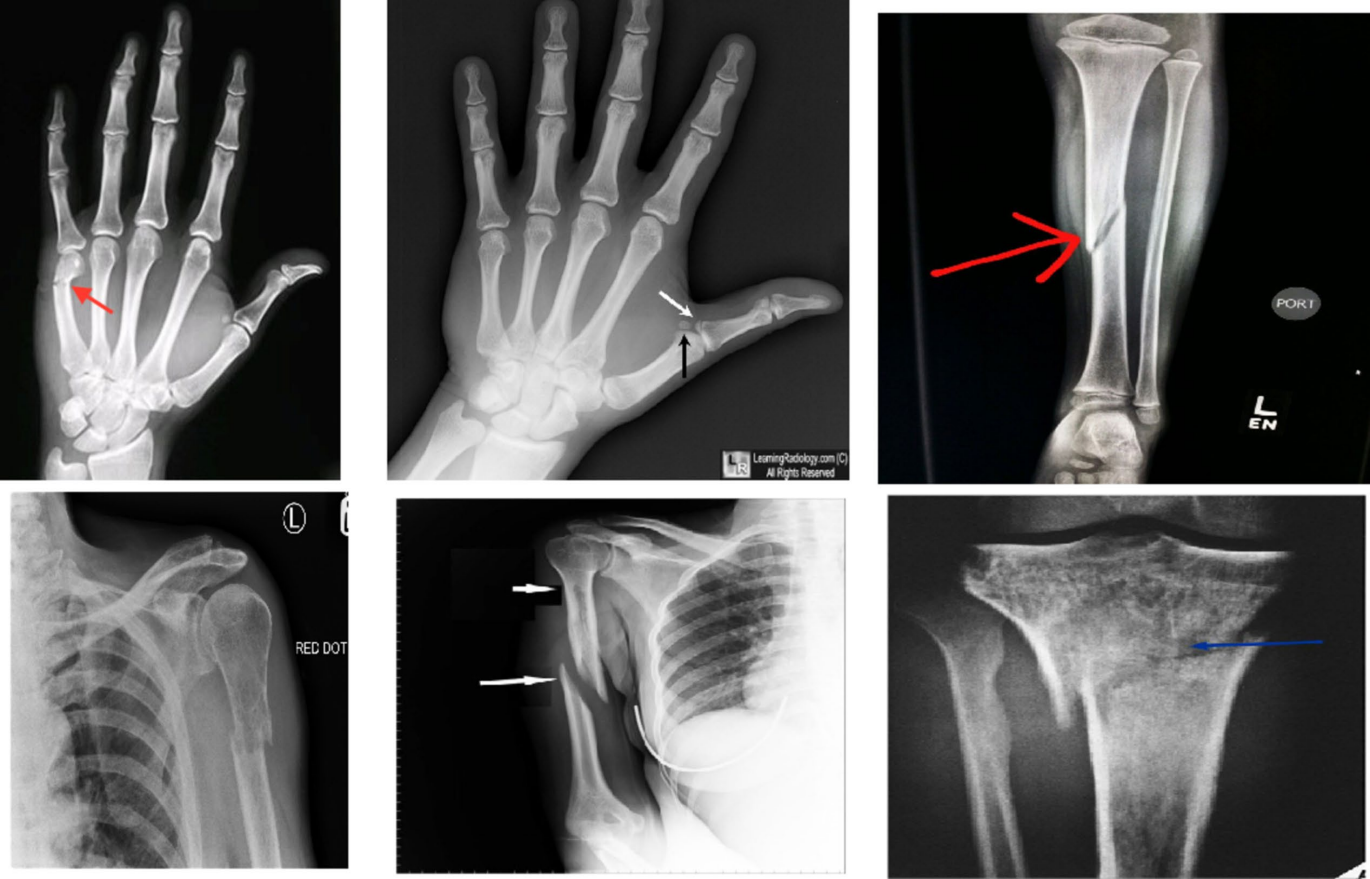
Shows bone fracture samples from the dataset.

In recent years, machine learning (ML) and deep learning (DL) methods have gained prominence in real-time medical analysis (4, 5). Various deep convolutional neural network (CNN) models have demonstrated success across multiple applications (6, 7). Bone fractures, as common injuries, necessitate immediate diagnosis. Although medical imaging system was sue to detect fractures, these images can be time-consuming, prone to error, and dependent on the clinician’s expertise (8, 9). Artificial intelligence (AI) technologies offer the potential to automate diagnostic processes, improving the speed and accuracy of fracture detection (10–13).The research has been conducted on the application of AI to bone fracture identification (14, 15). The different types of bone fractures are illustrated in Figure 1.

The increasing interest in using ML, particularly DL algorithms (16, 17), for processing medical images has been evident in recent years. Unlike traditional methods, DL algorithms can automatically extract features from images (18, 19). Notably, these methods have been employed to analyze X-rays and CT images, assess bone mineral density (BMD), identify fractures, and recommend treatments. In practice, doctors spend considerable time and effort in manually locating fracture sites. The application of DL in computer vision has prompted many experts to explore solutions to problems in medical imaging. DL methodologies, CNNs have been successfully applied across various domains, including healthcare (20–22). DL, a subset of ML, specializes in the analysis the medical images. For instance, while physicians identify bone fractures through visual assessment of X-rays, DL algorithms used to train for performing similar diagnostic using comprehensive databases of bone imagery (23–25).

This current research used to enhance existing AI-based tools for detecting bone fractures using medical imaging. It focuses on studies from the past 10 years, offering a comprehensive evaluation of different AI models, their clinical applications, and the challenges associated with their practical application. Additionally, the review highlights areas where research is lacking and proposes directions for future studies. The methodology adheres to a rigorous framework to ensure a structured analysis of the existing literature (26).

Our study makes key contributions to automated bone fracture detection. First, we propose an enhanced DL architecture incorporating VGG16, ResNet152V2, and DenseNet201 for fracture detection in X-ray images, integrating an attention mechanism for focused analysis and dilated convolutions for multi-scale feature extraction. Second, our model preserves fine-grained details through skip connections, which are crucial for detecting subtle fracture lines across various anatomical regions. Finally, this work presents a promising step toward developing automated tools to assist radiologists, potentially improving medical system for diagnosing fracture bone.

## 2 Relevant literature

The integration of artificial intelligence (AI) into medical imaging has significantly transformed the diagnosis of bone fractures across various modalities. X-ray imaging, a foundational tool for identifying fractures, exemplifies this impact, as AI algorithms, particularly those based on deep learning, enhance the detection of bone injuries, improve the identification of subtle fracture patterns, and assist in recognizing early indicators of conditions such as osteoporosis. This technological synergy not only increases diagnostic accuracy but also streamlines workflows, enabling radiologists to prioritize complex cases more effectively. Additionally, Computed Tomography (CT), which provides detailed cross-sectional images of bone structures, benefits from AI applications that facilitate precise fracture detection, monitor healing progress, and aid in the assessment of associated injuries. Similarly, Magnetic Resonance Imaging (MRI) plays a critical role in evaluating bone and soft tissue injuries, with AI enhancing image quality and interpretation through improved segmentation and anomaly detection. This is particularly valuable for complex fracture cases where surrounding soft tissue damage may complicate diagnosis. Overall, these advancements in AI across X-rays, CT, and MRI significantly enhance the accuracy of fracture detection and optimize clinical workflows, thereby reshaping the landscape of orthopedic imaging.

Rajpurkar et al. (27) developed CheXNet, a 121-layer CNN capable of diagnosing pneumonia from chest X-rays with greater accuracy than physicians. Trained on over 100,000 samples, CheXNet highlights the effectiveness of DL application in medical imaging. Lin et al. (28) combined classification and regression trees (CART) and case-based reasoning (CBR) to create a model for detecting liver cancer. CART was employed to identify liver issues, followed by CBR to pinpoint specific abnormalities, demonstrating the diagnostic potentials of AI. Meanwhile, Dombi et al. (29) employed ANN to predict rib fractures, utilizing 20 input features to predict the duration of hospitalizations, length of stays in critical care units, survival rates, and mortality rates, demonstrating AI’s role in healthcare decision-making. The ANN demonstrated an accuracy of 98%, indicating the significant potential for AI in early diagnosis and medical care. Zhang et al. (30) integrated language comprehension with musculoskeletal image analysis through TandemNet. Their approach enhances the accuracy and comprehensibility of DL models by integrating textual and visual data in the analysis of medical reports. Ypsilantis and Montana (31) developed a recurrent neural network (RNN) that uses visual attention to focus on critical areas of an image for precise detection of bone fractures, achieving efficient performance.

Fu et al. (32) developed a method for analyzing medical images that preserves the three-dimensional texture and structure of the proximal femur, enhancing the visibility of fracture lines and assisting physicians in identifying more intricate fracture patterns. Yaqub et al. (33) employed an unvalidated ML technique to categorize unidentified ultrasound images of fetuses into distinct categories, focusing on regions with prominent anatomical features. Their model achieved high classification accuracy on an extensive collection of clinical ultrasound images. Some studies indicated that their methodology was initially trained on a bone imaging dataset prior to categorization.

Yang et al. (34) developed CNN models to assist in the identification and differentiation of intertrochanteric fractures. Their dataset was divided into two segments: training, which included 32,045 images, and testing, which consisted of 11,465 photographs. A cascade architecture CNN was utilized to obtain the region of interest (ROI), followed by another CNN for segmentation and analysis. In a distinct investigation, Haitaamar and Abdulaziz (35) used the UNet model to segment rib fractures to categorize CT scan images, with images sized at 128 × 128 × 333 pixels. Nguyen et al. (36) applied the YOLO 4 model for fracture localization, improving performance through data augmentation techniques. Wang et al. (37) developed a pyramid network for bone diagnostics using X-ray images, and Ma et al. (38) proposed a two-phase approach for bone fracture identification. First, the rapid R-CNN detected 20 fracture sites, followed by CrackNet for fracture categorization. A unique Parallel Net methodology for fracture classification was also established, improving upon the two-scale approach of Wang et al. (39).

## 3 Methodology

**Figure 2:**
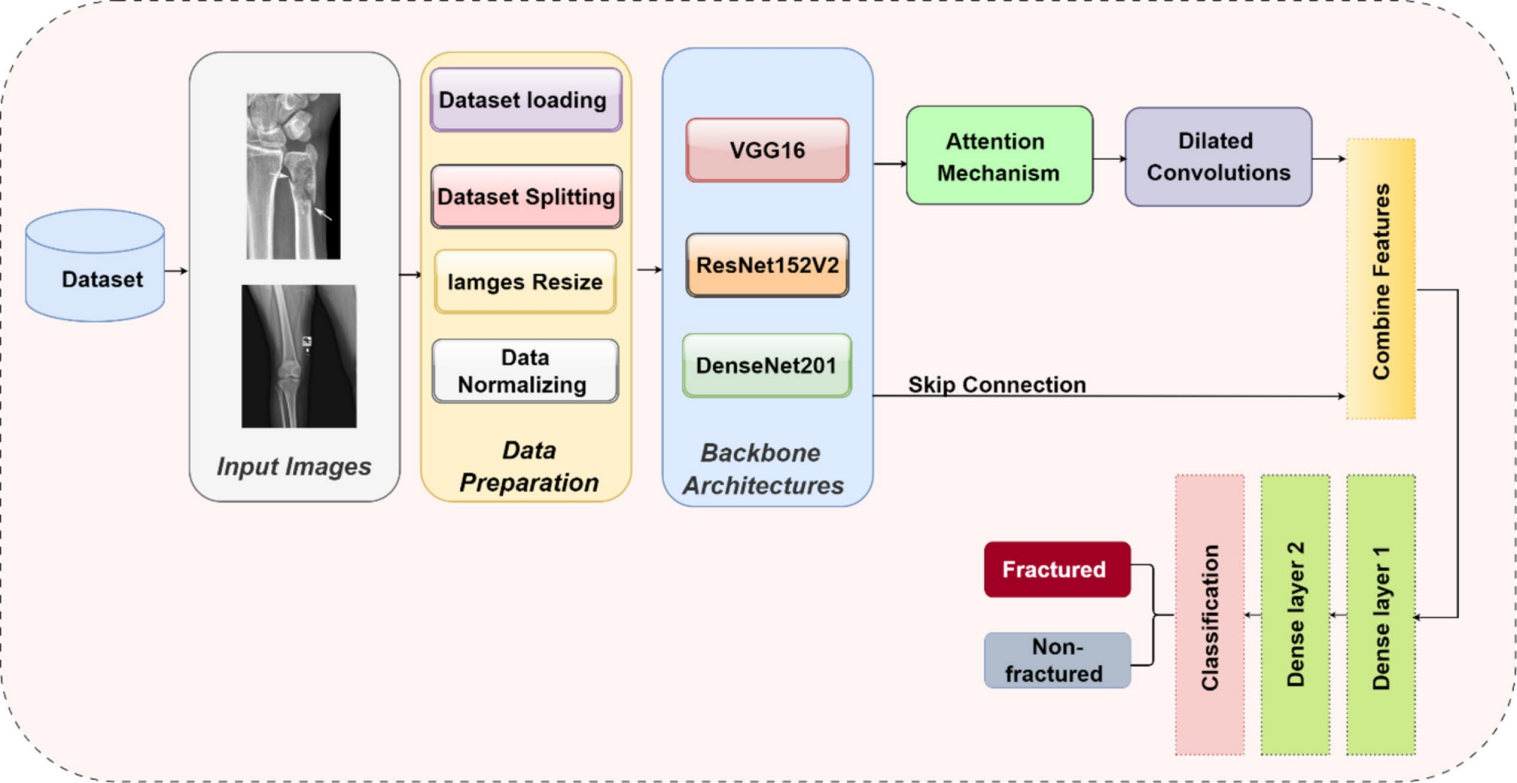
Architecture of the proposed bone fracture detection system.

The methodology in this study centers on developing and evaluating advanced DL models for the automatic detection of bone fractures using X-ray images. To accomplish this, we employed a DL approach that involved multiple stages, from data preprocessing to model training and evaluation. We used the bone fracture multi-region X-ray dataset, a comprehensive resource, to build and fine-tune custom models based on three well-established architectures, namely, VGG16, ResNet152V2, and DenseNet201. Each model was modified and optimized for the specific task of bone fracture detection, incorporating enhancements such as attention mechanisms and dilated convolutions to improve performance. Figure 2 displays the architecture of the proposed bone fracture detection system.

### 3.1 Dataset

**Figure 3:**
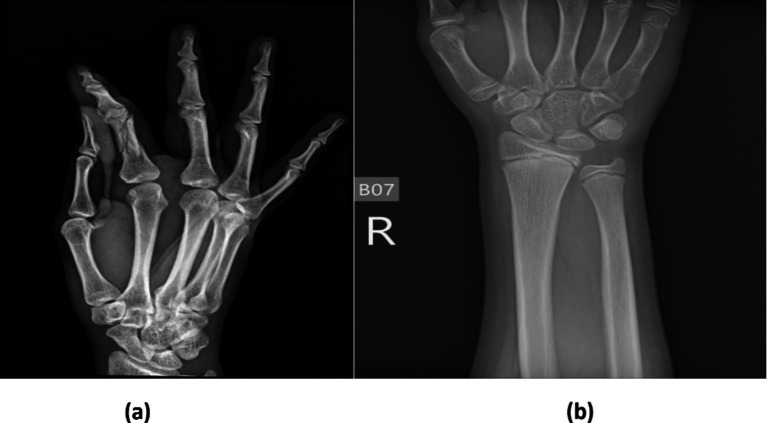
Sample images of (a) fracture and (b) non-fracture.

This research utilized the bone fracture multi-region X-ray dataset from Kaggle, comprising a total of 10,580 radiographic images. These images span various anatomical regions, including the lower limbs, upper limbs, lumbar spine, hips, and knees, and are categorized into fractured and non-fractured cases. The balanced distribution of the dataset makes it ideal for training DL models to detect fractures. The classes of bone fracture images are presented in Figure 3.

### 3.2 Preprocessing

**Figure 4:**
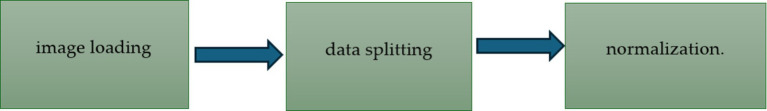
Preprocessing steps.

Preprocessing involved several key steps to prepare the dataset for model training, including image loading, data splitting, and normalization. The preprocessing steps of the bone fracture detection system are presented in Figure 4.

#### 3.2.1 Data splitting

**Figure 5:**
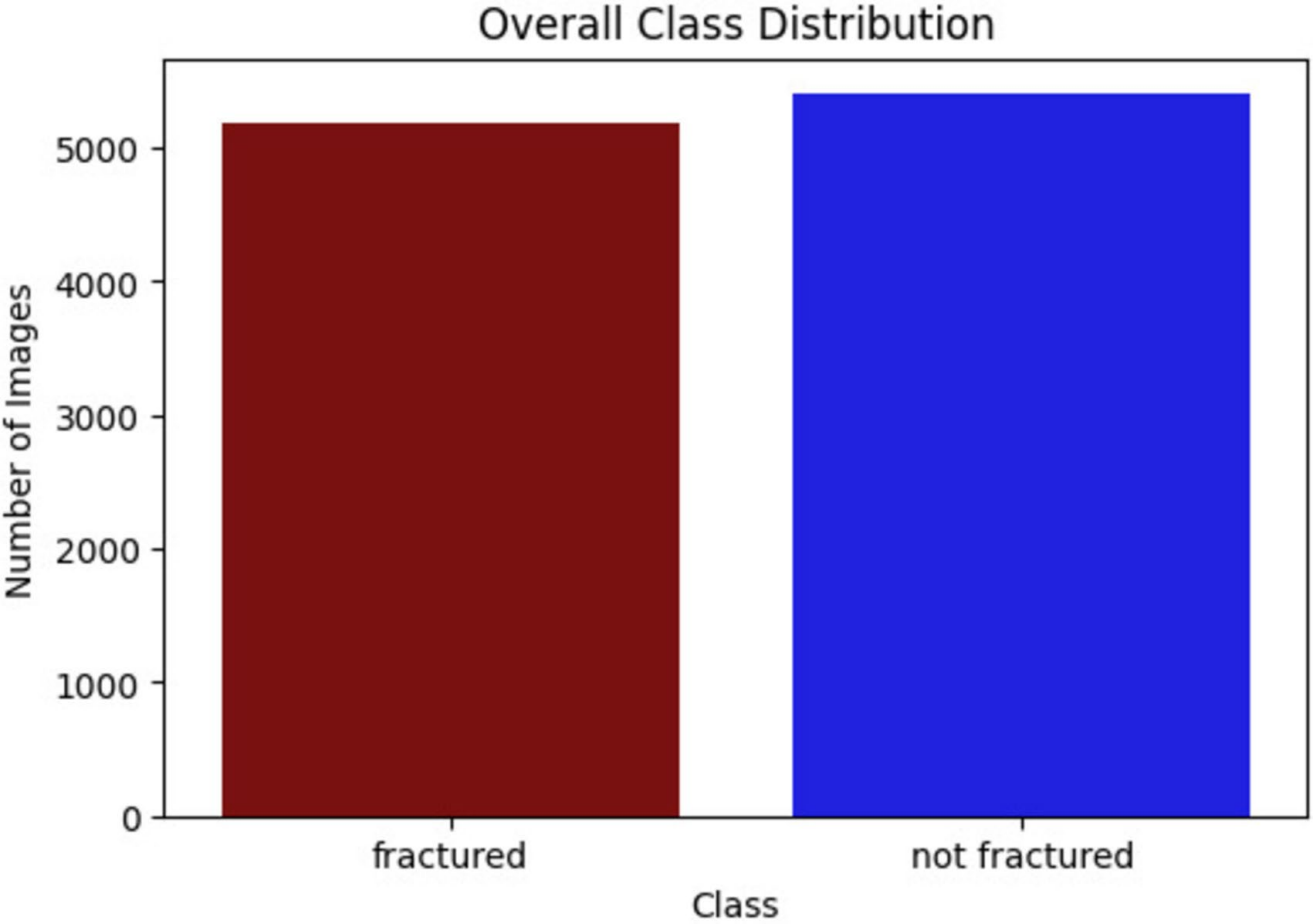
Classes of dataset.

The bone fracture multi-region X-ray dataset is divided into 7,406 training images, 2,115 test images, and 1,060 validation images, following a 70–20-10 split. This balanced distribution facilitates effective training, testing, and fine-tuning of the models across various anatomical regions, ensuring robust fracture detection. Figure 5 shows the number of images for each class.

### 3.3 Model architectures

Our research introduces novel enhancements to established DL architectures specifically tailored for the challenging task of bone fracture detection. We developed three models based on VGG16, ResNet152V2, and DenseNet201, incorporating innovative elements to enhance performance and address the unique challenges of medical image analysis.

#### 3.3.1 Common enhancements across models

To optimize the performance of the VGG16, ResNet152V2, and DenseNet201 architectures for bone fracture detection, we implemented four critical enhancements: an attention mechanism, dilated convolutions, skip connections, and global average pooling (GAP). These modifications were specifically designed to tackle the unique challenges of fracture detection in X-ray images.

#### 3.3.2 Attention mechanism

An attention mechanism was incorporated for focusing on key ROI within the X-ray images. The attention block processes input feature maps using both GAP and global max pooling methods. The resulting vectors are concatenated and subsequently processed using a shared multi-layer perceptron (MLP) with a bottleneck structure comprising two dense layers with ReLU activation. A sigmoid activation function is applied to generate attention weights, which are subsequently multiplied element-wise with the original input. This mechanism helps the model emphasize features that are most relevant to fracture detection, potentially replicating the targeted analysis conducted by radiologists.

#### 3.3.3 Dilated convolutions

To capture features at multiple scales, we implemented two dilated convolution blocks. The first block uses 256 filters with a 3×3 kernel and a dilation rate of 2, while the second block uses 128 filters with a 3×3 kernel and a dilation rate of 4. Both blocks are followed by ReLU activation and batch normalization. Dilated convolutions expand the receptive field without increasing the number of parameters, reducing spatial resolution. This approach allows the model to consider both local details and broader context simultaneously, which is crucial for detecting fractures of varying sizes and types.

#### 3.3.4 Skip connection

A skip connection was implemented to preserve fine-grained details. The output from the attention block is merged with the output from the second dilated convolution block via element-wise addition. When necessary, a 1×1 convolution is used to adjust channel dimensions before the addition. This integration mitigates the vanishing gradient problem in deep networks and helps the model retain important low-level features, which are critical for detecting subtle fracture lines.

#### 3.3.5 Global average pooling

GAP was applied after the skip connection to reduce the spatial dimensions. Each feature map is condensed to a single value by averaging all spatial locations, resulting in an output shape of 1x1xC, where C is the number of feature maps. GAP acts as a form of regularization, decreases the number of parameters, and emphasizes identifying features rather than their exact spatial positions. This is especially useful when working with X-rays that may have slight variations in positioning.

#### 3.3.6 Integration of enhancements

These enhancements were systematically integrated into each model in the following sequence: the base model (VGG16/ResNet152V2/DenseNet201), followed by an attention block, dilated convolution blocks, skip connections, GAP, and finally, dense layers for classification. The dense layers consist of 128 and 64 units, followed by a single unit with a sigmoid activation function for binary classification. This enhancement sequence aims to create a model capable of focusing on relevant areas, capturing multi-scale features, maintaining important low-level information, and making decisions based on the overall presence of fracture-indicating features in X-ray images.

### 3.4 Backbone architectures

In our study, we utilized three prominent DL architectures as the backbone for our bone fracture detection models: VGG16, ResNet152V2, and DenseNet201. Each of these architectures offers distinct advantages in the domain of medical image analysis.

#### 3.4.1 VGG16

**Figure 6:**
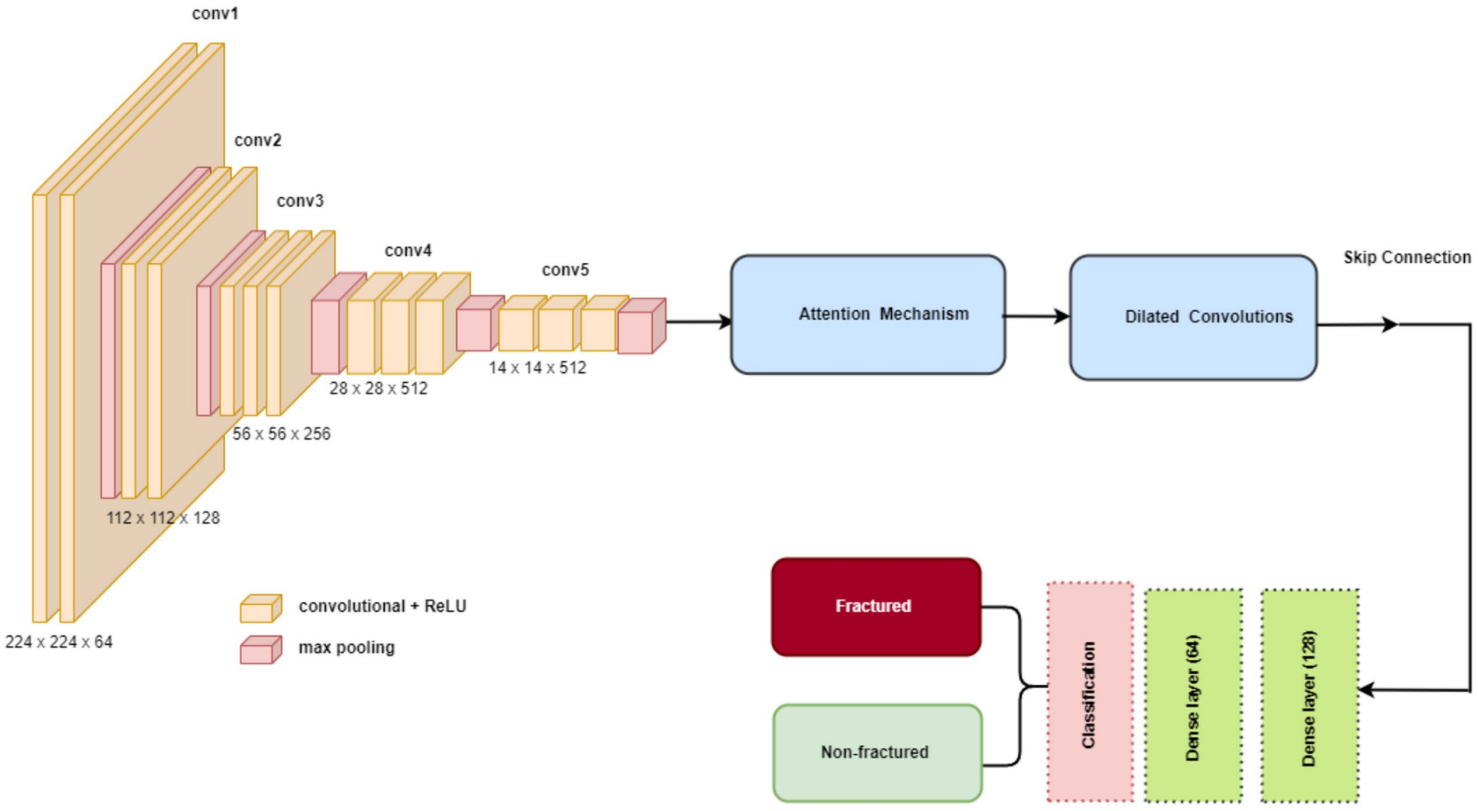
The VGG15 model.

VGG16 is known for its straightforward and consistent architecture, comprising a total of 16 layers, including 13 convolutional layers and three fully connected layers. A notable feature of VGG16 is its use of consistently small 3×3 convolutional filters throughout the network. This uniformity, combined with max-pooling layers for spatial dimension reduction, fosters an effective feature hierarchy. Despite having a large number of parameters (138 million), VGG16’s strength lies in its ability to capture hierarchical features effectively, making it well-suited for a variety of image recognition tasks, including medical imaging. The structure of VGG16 is shown in Figure 6. The key VGG16 parameters for developing the bone fracture detection system are presented in Table 1.

**Table 1:** VGG16 model architecture summary with layer types and output shapes.

#Layer name of VGG16	#Layer_Type of VGG16	#Output shape
input_1	InputLayer	(128, 128, 3)
vgg16_base	VGG16	(4, 4, 512)
attention_block	AttentionBlock	(4, 4, 512)
dilated_conv_1	Conv2D	(4, 4, 256)
dilated_conv_2	Conv2D	(4, 4, 128)
skip_connection	Add	(4, 4, 512)
global_avg_pool	GlobalAveragePooling2D	(512)
dense_1 system	Dense	(128)
dropout_1 system	Dropout	(128)
dense_2 system	Dense	(64)
dropout_2of system	Dropout	(64)
Output system	Dense	(Number of classes)

#### 3.4.2 ResNet152V2 model

**Figure 7:**
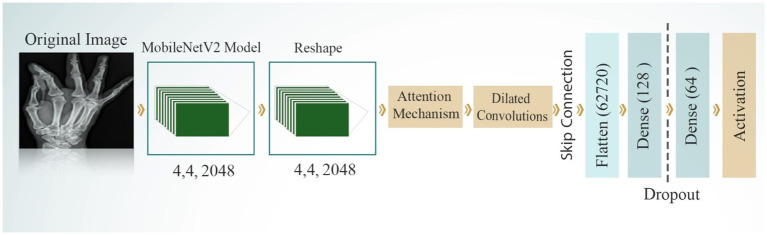
NesNet52v2 model for detecting bone fractures.

ResNet152V2, an improved version of the original ResNet, features a remarkable depth of 152 layers. Its primary innovation lies in the incorporation of residual blocks with skip connections, which enables the network to learn residual functions. This architecture effectively addresses the vanishing gradient problem, facilitating the training of very deep networks. ResNet152V2 includes several improvements over the original ResNet, such as batch normalization applied before convolutions to improve training stability, a bottleneck design (1×1, 3×3, 1×1 convolutions) in each residual block for increased efficiency, and pre-activation with ReLU activation applied before convolutions. Figure 7 displays the structure of the NesNet52v2 model for developing the bone fracture detection system. The parameters of ResNet152V2 that used to diagnosis bone fractures is presented in Table 2.

**Table 2:** ResNet152V2 output shapes.

#Layer name of NesNet52v2	#Layer_Type of NesNet52v2	#Output shape
input_1	InputLayer	(128, 128, 3)
resnet152v2	ResNet152V2	(4, 4, 2048)
attention_block	AttentionBlock	(4, 4, 2048)
dilated_conv_1	Conv2D	(4, 4, 256)
dilated_conv_2	Conv2D	(4, 4, 128)
skip_connection	Add	(4, 4, 2048)
global_avg_pool	GlobalAveragePooling2D	(2048)
dense_1 of system	Dense	(128)
dropout_1 of system	Dropout	(128)
dense_2 of system	Dense	(64)
dropout_2 of system	Dropout	(64)
Output of system	Dense	(Number of classes)

#### 3.4.3 DenseNet201 model

**Figure 8:**
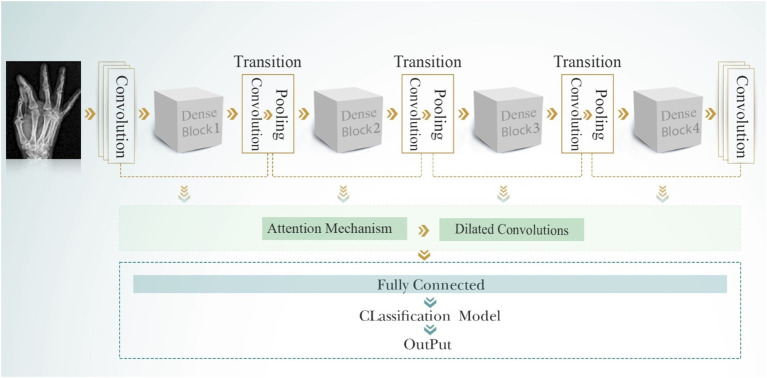
The DenseNet201 model.

DenseNet201 introduces an innovative dense connectivity structure, where each layer is directly connected to all preceding layers across dense blocks, resulting in a total of 201 layers. This feed-forward design allows for a more efficient information flow. Transition layers between the dense blocks are incorporated to reduce dimensionality and optimize performance. The network is characterized by its growth rate, which controls the number of feature maps added by each layer. This dense connectivity pattern enhances information flow and feature reuse, making DenseNet particularly effective for tasks that require fine-grained feature detection. Notably, DenseNet achieves high performance while using parameters more efficiently than traditional CNNs, as it encourages feature reuse throughout the network. Figure 8 shows the structure of the DenseNet201 model. The parameters of the DenseNet201 model for developing bone fracture detection are shown in Table 3.

**Table 3:** DenseNet201 model architecture summary with layer types and output shapes.

#Layer name of DenseNet201	#Layer_Type of DenseNet201	#Output shape
input_1 of system	InputLayer	(128, 128, 3)
densenet201	DenseNet201	(4, 4, 1920)
attention_block	AttentionBlock	(4, 4, 1920)
dilated_conv_1	Conv2D	(4, 4, 256)
dilated_conv_2	Conv2D	(4, 4, 128)
skip_connection	Add	(4, 4, 1920)
global_avg_pool	GlobalAveragePooling2D	(1920)
dense_1 of system	Dense	(128)
dropout_1 of system	Dropout	(128)
dense_2	Dense	(64)
dropout_2	Dropout	(64)
Output	Dense	(Number of classes)

## 4 Experimental results

This section details the experimental outcomes of our study, which evaluated the performance of three custom DL models—VGG16, ResNet152V2, and DenseNet201—designed for automatic bone fracture detection from X-ray images.

### 4.1 Environment setup

The results obtained in this study were generated using equipped with an eighth-generation Intel Core i7 processor, 16 GB of RAM. The TensorFlow framework (36) was employed for model development. These hardware and software configurations for ensuring the efficient training of the DL models.

### 4.2 Evaluation metrics

To assess our models’ performances, we evaluated them using the confusion matrix. Altogether, these metrics provided a thorough evaluation of the models’ classification performances. Equations 1–4 shows the evaluation metrics equation.


(1) $$\mathrm{Accuracy}=\frac{TP+TN}{FP+FN+TP+TN}\times 100$$

(2) $$F1-\mathrm{score}=2*\frac{\mathrm{precision}\times \mathrm{Recall}\;}{\mathrm{precision}+\mathrm{Recall}\;}\;x100\%$$

(3) $$\mathrm{Sensitivity}=\;\frac{\mathrm{True}\;\mathrm{Positives}\;}{\mathrm{True}\;\mathrm{Positives}+\mathrm{False}\;\mathrm{Positives}\;}x100\%$$

(4) $$\mathrm{Specificity}=\frac{\mathrm{True}\;\mathrm{Negatives}}{\mathrm{True}\;\mathrm{Negatives}+\mathrm{False}\;\mathrm{Negatives}}x100\%$$

### 4.3 Models’ performances

The models based on VGG16, ResNet152V2, and DenseNet201 were trained on the binary classification dataset for bone fractures to differentiate between fractured and non-fractured bone images. All three models employed the Adam optimizer with a learning rate of 0.0001 and a batch size of 16. The training was capped at a maximum of 50 epochs, with early stopping implemented after five consecutive epochs of no improvement in validation loss. Additionally, when validation loss of proposed model is remained unchanged for three epochs, the learning rate was reduced in to 0.2, with a minimum threshold of 1e-6. These optimization strategies were employed to ensure efficient training and to prevent overfitting. The specific hyperparameters for each model are shown in Table 4.

**Table 4:** Hyperparameters of each model.

#Hyperparameter	#Values of system
Optimizer_Function_proposed system	Adam
Learning_Rate_proposed system	0.0001
Loss_Function_proposed system	Binary cross-entropy
Batch_Size_proposed system	16
Number_Epochs	50
Early stopping patience	5
Reduce LR factor	0.2
Reduce LR patience	3
Min LR	1e-6

#### 4.3.1 ResNet152V2’s performance

The ResNet152V2 model was trained on bone fracture images for a binary classification dataset with a maximum of 50 epochs, but the training process was halted early after 22 epochs due to a lack of improvement in the validation loss. Utilizing the Adam optimizer with a learning rate of 0.0001 and a batch size of 16, as detailed in Table 5, the RestNet52V2 achieved an accuracy of 92.15%, an F1-score of 92.35%, a sensitivity of 92.86%, and a specificity of 91.84%.

**Table 5:** Results for diagnosis bone fractures using ResNet152V2 model.

Class name	Precision (%)	Recall (%)	F1-Score (%)	Support/validation
Fractured	92	91	92	1,036
Not fractured	92	93	92	1,079
Accuracy			92	2,115
Macro_Avg_over system	92	92	92	2,115
Weighted_Avg_over system	92	92	92	2,115

**Figure 9:**
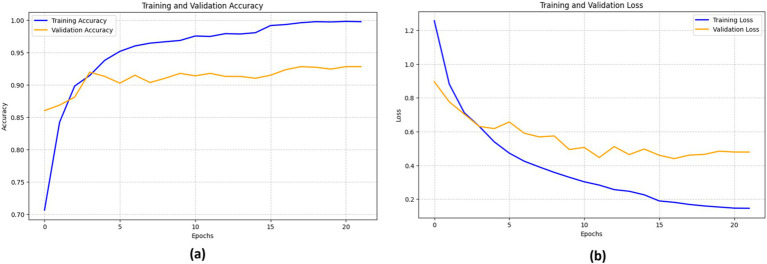
Performance of the ResNet152V2 model in terms of (a) accuracy and (b) loss.

Figure 9 illustrates the performance of the ResNet152V2 model. The training accuracy (blue line) consistently improves, nearing 100%, while the validation accuracy (orange line) rises rapidly during the initial epochs, peaking at around 90% before stabilizing.

The loss curves show a sharp decline in training loss (blue line) as the epochs advance. However, the validation loss (orange line) demonstrates a comparable but less pronounced drop. At about epoch 5, the validation loss starts fluctuating, suggesting potential issues with the model’s generalization capability on unknown data. This variability indicates that the model may benefit from fine-tuning to enhance its resilience.

**Figure 10:**
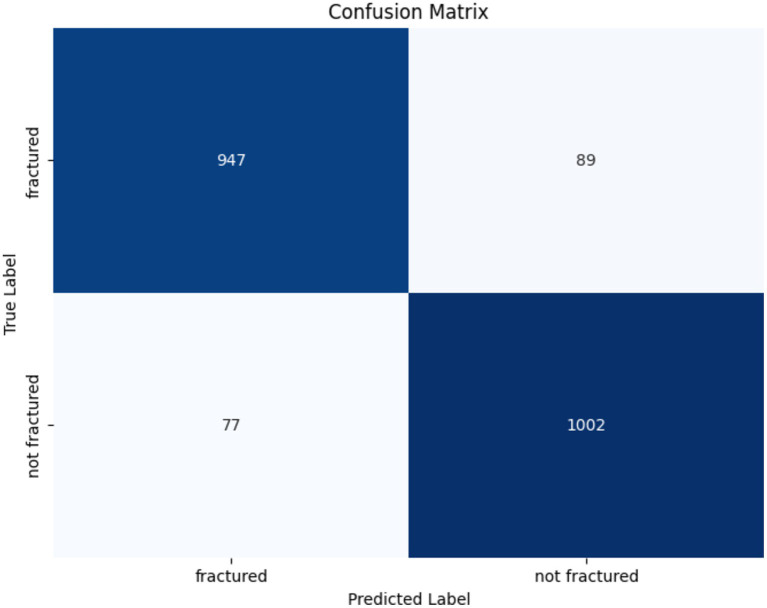
Confusion matrix of the ResNet152V2.

The confusion matrix illustrating the performance of the ResNet152V2 model for bone fracture classification is shown in Figure 10. The algorithm accurately identified 947 fractured instances (TP) and 1,002 non-fractured cases (TN). However, it erroneously categorized 89 fractured instances as non-fractured (FN) and 77 non-fractured cases as fractured (FP). These findings show that while the model demonstrates strong efficacy in both detecting and excluding fractures, there is little opportunity for enhancement in minimizing misclassifications, especially FNs, which are crucial in medical diagnostics.

#### 4.3.2 VGG16’s performance

The VGG16-based model’s training ended after 20 epochs due to early stopping, achieving an accuracy of 96.55%, which reflects strong performance in distinguishing between fractured and non-fractured images. Its F1-score of 96.64% underscores a good balance between precision and recall. The model’s recall (sensitivity) reached 97.31%, showing its effectiveness in accurately identifying fractured cases. Meanwhile, the precision (specificity) was 95.98%, indicating its effectiveness in reducing FP. Table 6 shows the results of the VGG16 model’s performance.

**Table 6:** Results for diagnosis bone fractures using VGG16 model.

Class name	Precision (%)	Recall (%)	F1-score (%)	Support/validation
Fractured	97	96	96	1,036
Not fractured	96	97	97	1,079
Accuracy			97	2,115
Macro_Avg_over system	97	97	97	2,115
Weighted_Avg_over system	97	97	97	2,115

**Figure 11:**
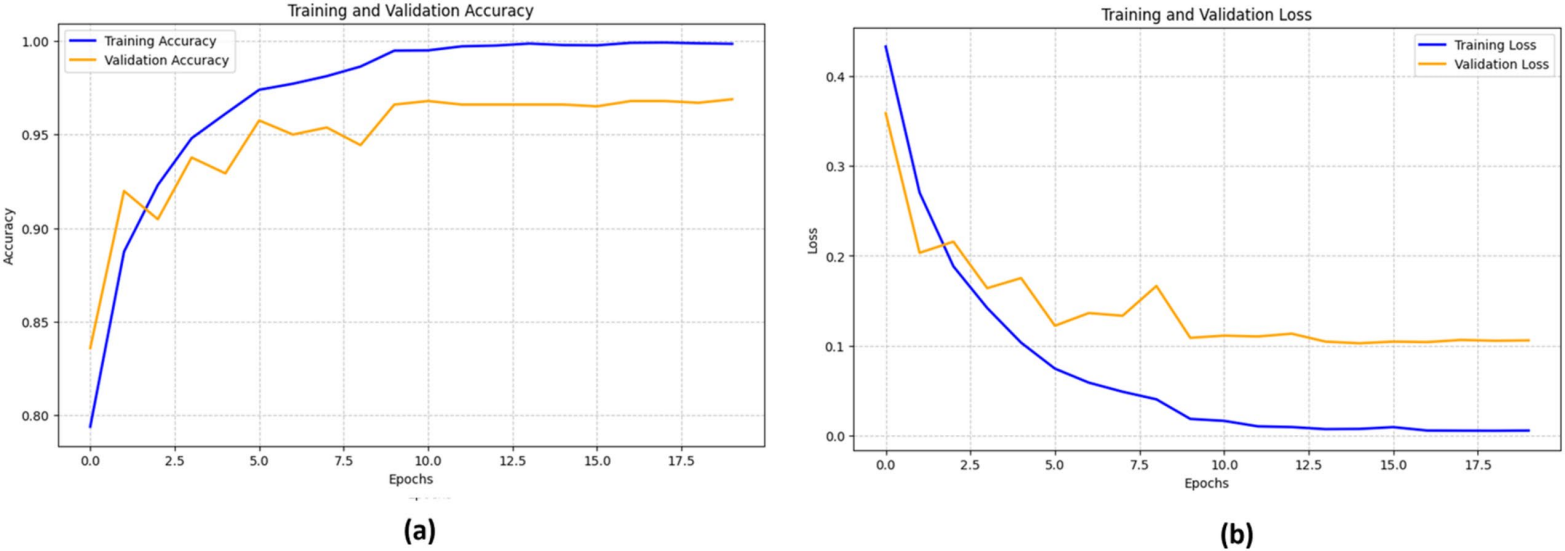
Performance of the VGG16 model in terms of (a) accuracy and (b) loss.

Figure 11 depicts the effectiveness of the VGG16 model in classifying bone fractures. The training accuracy (blue line) increases significantly, nearing 100%. This signifies the model’s excellent assimilation of the training data. The validation accuracy (orange line) exhibits a similar pattern, reaching a maximum of about 95% before stabilizing after several epochs.

The training loss (blue line) shows a steady decline as the model continues to learn, while the validation loss (orange line) initially reduces before stabilizing with slight variations. These consistent validation loss values suggest that the model is not significantly overfitting, although the minor variations imply room for further fine-tuning to optimize performance.

**Figure 12:**
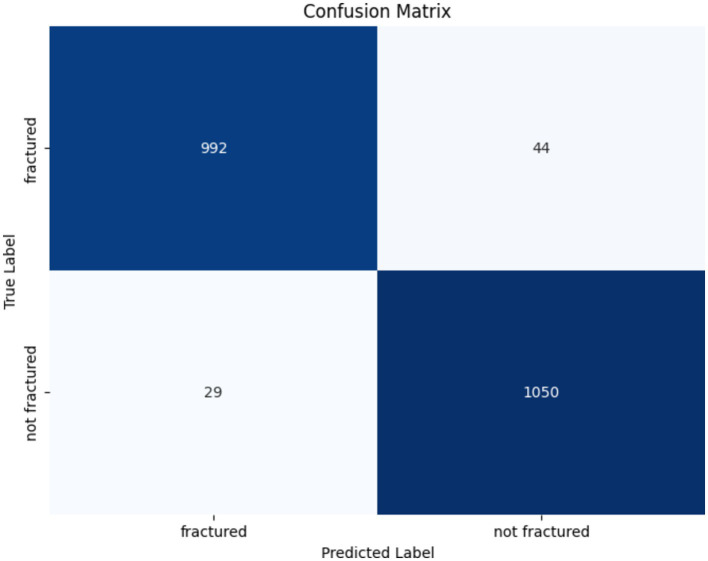
Confusion matrix of the VGG16 model.

The confusion matrix for the VGG16 model in identifying bone fractures indicates robust performance, as presented in Figure 12. The algorithm precisely predicted 992 fractured and 1,050 non-fractured instances. Notably, it misclassified 44 fractured occurrences as non-fractured and 29 non-fractured instances as fractured. The VGG16 model has great accuracy and recall, with few false positives and false negatives, hence confirming its efficacy in differentiating between fractured and non-fractured bones.

#### 4.3.3 DenseNet201’s performance

The DenseNet201-based model’s training concluded after 39 epochs due to early stopping, with a final accuracy of 97.35%, demonstrating its robust performance in distinguishing between fractured and non-fractured images. The F1-score of 97.41% highlights a balanced trade-off between precision and recall, demonstrating the model’s efficacy. With a sensitivity (recall) of 97.78%, the model effectively identified fractured cases, and its specificity (precision) of 97.06% reflects its capability to minimize false positives. These results underscore the reliability of the DenseNet201 model in bone fracture detection tasks. The key results of DenseNet201 are summarized in Table 7.

**Table 7:** Results for diagnosis bone fractures using DenseNet201 model.

Class name	Precision (%)	Recall (%)	F1-score (%)	Support/validation
Fractured	98	97	97	1,036
Not fractured	97	98	97	1,079
Accuracy		97		2,115
Macro_Avg_over system	97	97	97	2,115
Weighted_Avg_over system	97	97	97	2,115

**Figure 13:**
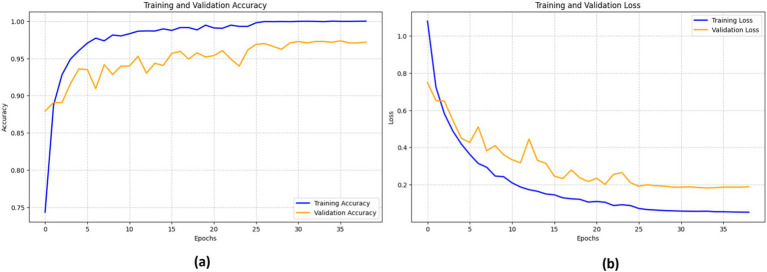
Performance of the DenseNet201 model.

The efficacy of the DenseNet201 model in identifying bone fractures is demonstrated in Figure 13. The training accuracy (blue line) shows a sharp increase during the first epochs, eventually stabilizing at approximately 98%. The validation accuracy (orange line) follows a similar pattern, though it remains somewhat lower than the training accuracy, reaching a peak of around 96%.

The training loss (blue line) and validation loss (orange line) both exhibit a significant decrease in the early epochs. This signifies that the model rapidly reduces errors during the training process. However, after about 15 epochs, the validation loss begins to fluctuate, while the training loss consistently declines, indicating a probable onset of overfitting.

**Figure 14:**
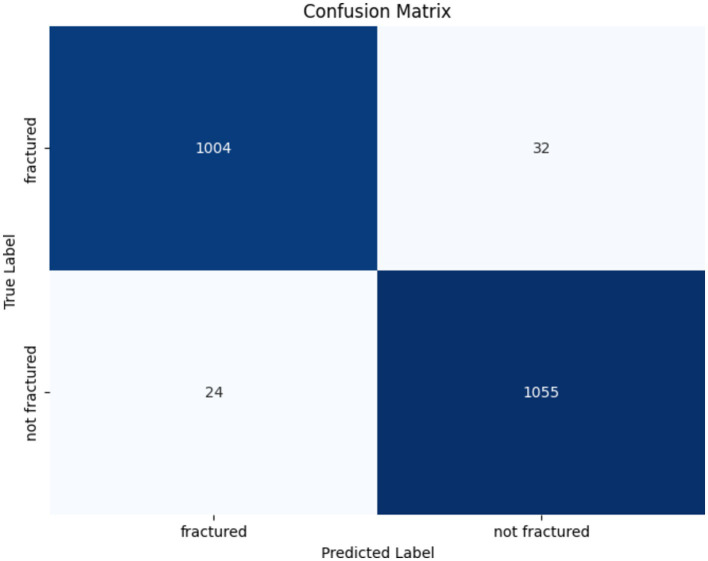
Confusion matrix of the DenseNet201 model.

The confusion matrix of the DenseNet201 model is presented in Figure 14. Out of the 1,036 fractured instances, the model correctly categorized 1,004, with only 32 cases being misclassified as non-fractured. Similarly, among 1,079 non-fractured examples, the model accurately classified 1,055, with only 24 misclassified as fractured. These findings emphasize the DenseNet201 model’s exceptional accuracy and minimal misclassification rates, underscoring its reliability in differentiating between fractured and non-fractured bones in medical imaging applications.

## 5 Discussion and comparative analysis

In recent years, bone fracture diagnosis and classification have garnered significant research interest due to the increasing need for reliable solutions. Is it possible to identify and treat every fracture in the human body? This work aimed to simplify fracture diagnosis and accelerate the diagnostic process by leveraging DL models to examine bone images.

Three modified DL models—DenseNet 201, ResNet 152 V2, and VGG16—were trained for bone fracture diagnosis and evaluated based on four key criteria: accuracy, F1-score, sensitivity (recall), and specificity. This comparative analysis provided deeper insights into each model’s performance.

DenseNet 201 emerged as the most consistent model, achieving a maximum accuracy of 97.35%. This high level of accuracy underscores the model’s precision in distinguishing between fractured and non-fractured bone scans. With an F1 score of 97.41%, DenseNet201 successfully balances accuracy and recall, minimizing both false positives and false negatives. Additionally, the model’s sensitivity of 97.78% indicates its strong ability to consistently detect actual fracture cases, while its 97.06% specificity reflects its effectiveness in reducing false positives.

The VGG16 model also performed notably well, with an accuracy of 96.55% and an F1-score of 96.64%. With a specificity of 95.98%, VGG16 showed relatively poor performance, indicating a significantly elevated incidence of false positives. Although the model demonstrated superior efficacy in fracture recognition (97.31% sensitivity) compared to DenseNet201, its specificity for fractures was much lower.

ResNet152V2 underperformed compared to the other models, with an F1-score of 92.35%, accuracy of 92.15%, and sensitivity of 92.86%. Its relative inability to adequately minimize false positives rendered its specificity of 91.84% unsuitable for this specific task.

Table 8 distinctly highlights the performance metrics of the DenseNet 201, ResNet152V2, and VGG16 models in terms of accuracy, F1-score, sensitivity (recall), and specificity (precision). While all three models demonstrated the capability to identify bone fractures, DenseNet201 was the most reliable and precise, exhibiting superior balance across all critical performance metrics. VGG16 followed closely in second place, while ResNet152V2, despite its functionality, was less suited for medical image classification in this context, exhibiting limitations in both accuracy and recall. We provide a comprehensive performance analysis encompassing all three versions.

**Table 8:** Performance of the DL model.

Model name	Accuracy (%)	F1-score (%)	Sensitivity (%)	Specificity (%)
ResNet152V2	92.15	92.35	92.86	91.84
VGG16	96.55	96.64	97.31	95.98
DenseNet201	97.35	97.41	97.78	97.06

**Figure 15:**
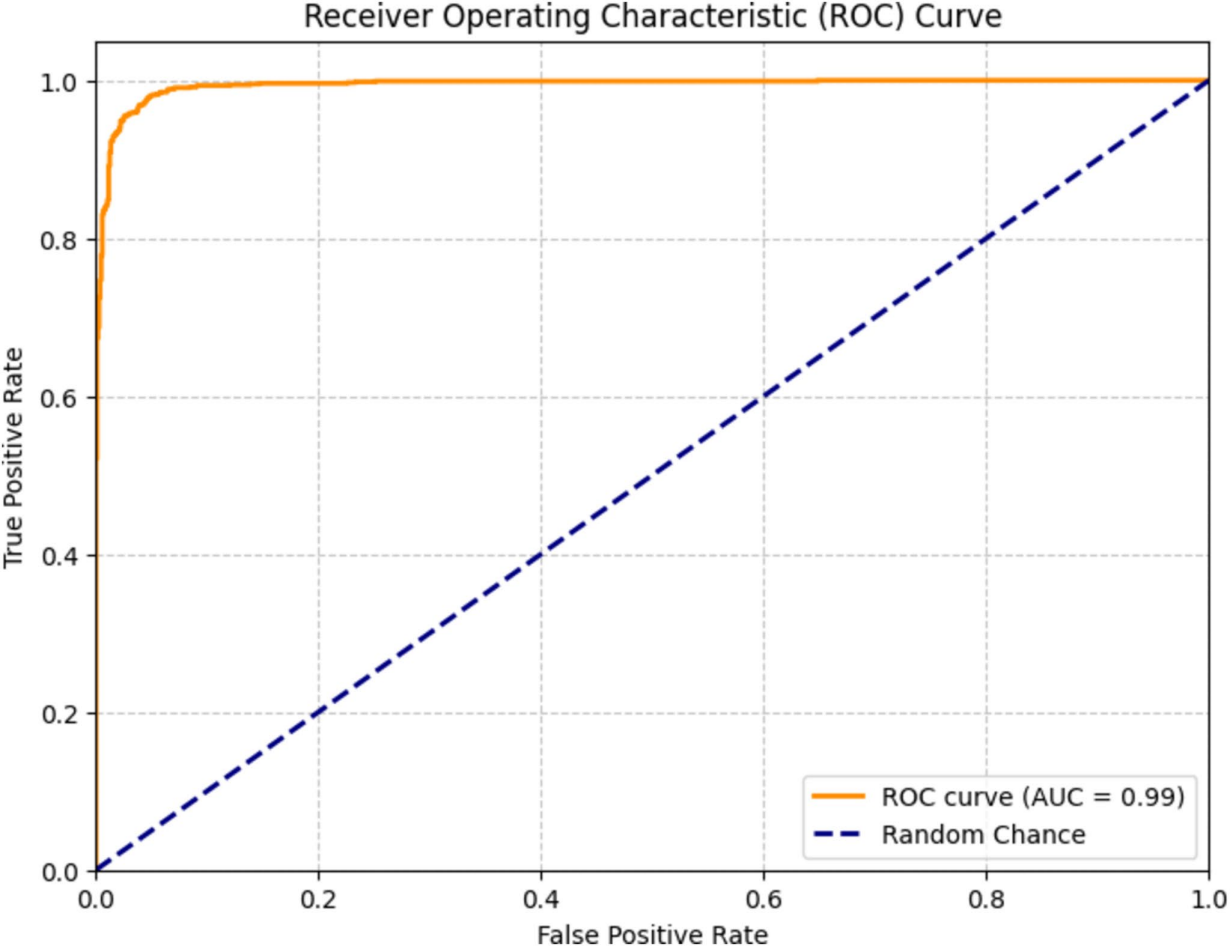
ROC of DenseNet201.

Figure 15 depicts a receiver operating characteristic (ROC) curve used to assess the efficacy of a DenseNet201 classification model for bone fracture diagnosis. The graph presents the true positive rate (TPR) in relation to the false positive rate (FPR) across various threshold configurations. The orange line represents the ROC curve of the model, showcasing its ability to differentiate between classes. The model scored of 0.99, indicating exceptional performance in accurately classifying fractures.

DenseNet201 outperformed ResNet152V2 and VGG16 mainly due to its dense connectivity structure, which connects each layer to all preceding layers. This architecture allows for more efficient feature reuse and better gradient flow, helping the model capture both fine details and larger patterns. In the context of medical images like X-rays, where subtle fractures may be difficult to detect, this enhanced feature extraction made DenseNet201 particularly effective. Moreover, DenseNet201 uses fewer parameters compared to the other models, despite its depth. This efficiency reduces the risk of overfitting, which is crucial when working with medical image datasets that can be limited in size. The ability to maintain high performance with fewer parameters gave DenseNet201 a clear advantage in our experiments. Table 9 shows comparison of diagnosis system based on DL models for detecting and classifying bone fracture.

**Table 9:** comparison results.

References	Model	Accuracy
Karimunnisa et al. (40)	BPNN	91%
Abbas et al. (41)	R–CNN	94%
Castro-Gutierrez et al. (42)	SVM	80%
Proposed system	DenseNet201	97%

## 6 Limitations and future work

Despite the promising results achieved in bone fracture detection, several limitations warrant acknowledgment. The primary constraint lies in our dataset’s composition, which, while substantial, could benefit from greater diversity in fracture types to enhance model generalizability. Furthermore, our current approach’s exclusive focus on X-ray imaging modalities, though practical, may limit the system’s broader clinical applicability. To address these limitations and advance the field, we propose several directions for future research. First, architectural enhancements to ResNet152V2 and VGG16 could incorporate transformer-based attention mechanisms, potentially improving the detection of subtle fractures and focusing on critical regions within X-ray images. We also suggest exploring alternative architectural designs for deeper feature extraction and efficient processing, aiming to optimize the balance between model complexity and performance. Image processing improvements represent another promising avenue. Implementing advanced enhancement techniques, including contrast adjustment and noise reduction, could significantly improve fracture visibility and, consequently, detection accuracy. Additionally, integrating segmentation techniques could enable precise isolation of fracture regions, enhancing both model precision and diagnostic utility. Finally, expanding the scope of imaging modalities beyond X-rays to include CT and MRI scans could broaden the system’s clinical applications. This multi-modal approach, combined with a more diverse dataset, would strengthen the model’s generalizability and practical value in various medical settings.

## 7 Conclusion

Fracture patients often present as emergencies and can be inaccurately diagnosed using radiologic imaging. A growing body of research employs AI methodologies to assist in fracture identification and complement physician diagnoses. DL methods have been established as crucial tools in disease detection and treatment, and researchers are investigating cutting-edge technologies to improve healthcare processes. The automation of bone fracture detection and categorization remains a key area of research. Traditional methods for evaluating lower leg bone fractures have faced challenges in accurately detecting and locating fractures.

To address these issues, we proposed a transfer learning model utilizing VGG16, ResNet152V2, and DenseNet201 for fracture identification and classification. We assessed this model using a standard dataset of 10,581 images, achieving an overall accuracy of 97% in both classification and detection. Our research demonstrates that the presented technique is not only simple but highly effective, proving to be beneficial for dynamic fracture detection and classification. This enables physicians and radiologists to handle a greater number of patients while reducing their workload. The proposed strategy enhances outcomes, improves runtime performance, and augments detection quality compared to state-of-the-art solutions. This article offers clinicians valuable insights into recent advancements in AI-driven fracture diagnosis by reviewing existing research in the field.

## Data Availability

The original contributions presented in the study are included in the article/supplementary material, further inquiries can be directed to the corresponding authors.
